# Lived Experiences of Mental Health Recovery in Persons of Culturally and Linguistically Diverse (CALD) Backgrounds within the Australian Context

**DOI:** 10.1007/s40737-022-00319-y

**Published:** 2022-12-05

**Authors:** E. Levy-Fenner, E. Colucci, S. McDonough

**Affiliations:** 1 Department of Psychology, Middlesex University, London, UK; 2 Global and Cultural Mental Health Unit, The University of Melbourne, Melbourne, Australia; 3 Victorian Transcultural Mental Health, St. Vincent’s Hospital, Melbourne, Australia; 4 Australian Institute for Primary Care and Ageing, La Trobe University, Melbourne, Australia

**Keywords:** Lived experience, Recovery, Mental health, CALD, Ethnic minority, Digital storytelling, Visual methods

## Abstract

Lived experience research related to mental health recovery is advancing, but there remains a lack of narrative material from the perspectives of people from under-represented, non-dominant cultural backgrounds in this domain. This study aimed to explore the lived experiences of mental health recovery in people of culturally and linguistically diverse (CALD) backgrounds in the Australian context. The current study involved a secondary analysis of audio and visual data collected during the digital storytelling project *Finding our way* in Melbourne, Australia. Thematic analysis was used to understand the lived experience narratives of nine participants in relation to mental health recovery. Five themes were identified through an iterative process of analysis, including *Newfound opportunities and care*, *Family as key motivators and facilitators*, *Coping and generativity*, *Cultivating self-understanding and resilience*, and *Empowerment through social engagement*. First person lived experience narratives offer deep insight into understanding the ways in which individuals of marginalised communities conceptualise and embody recovery. These findings further the literature and understanding on how to better serve the needs of people with mental health challenges from CALD communities through informed knowledge of what may be helpful to, and meaningful in, individuals’ recoveries.

## Introduction

Recovery can be thought of as a “redefinition of oneself in light of lived experience” and as having a newfound sense of balance and wellbeing (Pelletier et al., 2020, p. 2). Literature focused on the gulf between *clinical* and *personal* recovery (Slade, 2009) is substantial, and the development of understanding of the lived experience of recovery and recovery-oriented care (Davidson et al., 2021) has been furthered by the mental health consumer movement (Deegan, 1996, 2007). A salutogenic approach (Antonovsky, 1979) to mental health recovery is well established and models of positive functioning have been developed and extended in recent years (Provencher & Keyes, 2013). While more people with lived experience of mental illness are continuing to share their stories of recovery (Drake & Whitley, 2014), there is a dearth of research related to under-represented, non-dominant cultural narratives, often referred to as CALD and ethnic minority communities, and the experiences of people in this domain (Tang, 2019).

Antonovsky recognised the significance of cultural stress, something he termed the “ethnic… handicap” (Antonovsky, 1967, p.393) and the struggle for minority groups to be seen and heard within dominant cultural narratives. Nevertheless, discussions around the concept of recovery often fail to recognise the powerlessness that comes from cultural imperialism (Bonnington & Rose, 2014), discrimination against those involved as mental health service users, and the complex ways in which processes of recovery are embedded in, and sometimes also impeded by, social structures and forces (Morrow & Weisser, 2012). Rose (2014) contended that while recovery was once about liberation, it has come to be “instrumentalised and mainstreamed… with our neoliberal present” (p. 217). As such, current conceptualisations of recovery may diminish the importance of social relations, be intolerant of difference, and have the potential to neglect epidemiological evidence demonstrating that social and health inequalities, as well as overt and covert racism, can both impact and lead to mental distress (Bhurga et al. 2010, 2021; Bogic et al., 2015; Edge & Bhugra, 2016; Fernando, 2017; Kirkbride & Jones, 2010; Nazroo et al., 2020). Harper and Speed (2012) discussed how individualising recovery conceals the social and structural causes of great distress which then “impacts upon the potential for recovery to be used to explore more collective and political aspects of emotional distress” (p. 8). In addition, the authors reference Lemke (2001) as they reflected on the various governments and NGOs who frame recovery as a form of self-management through responsibilisation, which serves to reduce the impact of structural issues and moves to frame recovery as a dutiful act of self-care (Harper & Speed, 2012). Morrow and Weisser (2012) called for an intersectional social justice approach to recovery in which those with lived experience are not “co-opted by professionalism” (p. 29). Furthermore, an intersectional approach reframes “issues of difference”, from essentialist categories of race, gender, class, and sexuality to a relative construct or “organizing principles of a society that position members of various groups within its opportunity structure” (Talwar, 2010, p. 12).

### Medicalisation and Culture

Applications of a reductionist biomedical model and the entanglement of patriarchy, psychiatry, and colonialism impacts the ways in which mental health systems are built and maintained in various parts of the world (Bracken et al., 2021; Marsella, 2010; Pūras, 2017, 2020). Moreover, terminologies of dysfunction and disease stemming from the biomedical model may influence how people perceive themselves (Adame & Hornstein, 2006). Medicalisation often fails to recognise social context, alienates individuals in need of help, and entrenches systems of power that are ill-suited to localised contexts (Eaton, 2019; Hopper, 2007; Wong et al., 2021). Different cultures often conceptualise mental health in unique ways, and this will influence how individuals construct knowledge, express and present feelings and symptoms, and approach help-seeking (Procter et al., 2014; Talwar, 2010). Metzel (2009) highlighted that even definitions of mental illness are inconsistent, are influenced by socio-political forces, with there likely to be a series of factors that contribute to the development of illness. Undeniably, individuals are enmeshed in interdependent networks, and both illness and recovery will arise from and reflect these networks (Duff, 2016).

The Cultural Influences on Mental Health (CIMH) model (Hwang et al., 2008) is a conceptual paradigm that explores the impacts of culture on mental health. The model identifies six interrelated and non-exhaustive domains in which culture plays a role in the nuances of understanding mental health, including the phenomenology or expression of distress, coping styles, and help-seeking pathways. Manifestations of distress may be understood, communicated, and tolerated in varying ways, and are shaped and filtered by the sociocultural context, norms, and beliefs in which an individual is situated (Crowe, 2006; Hwang et al., 2008; Kaiser et al., 2013; Kleinman, 1978; Marsella, 1980). Neglecting these differences has the potential to maintain and reinforce oppressive power relations (Gómez-Carrillo et al., 2020; Jadhav, 2009) through “maintaining the parameters of normality…that reflects particular gender, culture and class biases” (Crowe, 2006, p. 125).

However, research does also suggest both dominant and minority groups value personal agency and being able to express themselves appropriately in working towards recovery (Brijnath, 2015; Casey & Webb, 2019; Kalathil et al., 2011; Milasan et al., 2020; Tang, 2019; Vansteenkiste et al., 2021). In a study of Chinese people utilising mental health services in the United Kingdom, “being able to make sense of their distress within their lifeworld experience” was important (Tang, 2019, p. 277). Lo (2010) in discussing the work of Mishler (1984) conceptualised the *lifeworld* as patients’ accounts of their “context-sensitive illness experiences… oriented to meanings, understanding, and a sense of groundedness in everyday life” (Lo, 2010, p. 486). This is important, as idioms used to express distress in minority populations are often different to those commonly known to health professionals in majority English-speaking nations (Bhavsar et al., 2019; Bhui & Black, 2010; Watson & Bhugra, 2020). Research suggests social alienation that can occur alongside the process of cultural adjustment, has the potential to produce chronic psychological difficulties, and that grief reactions and bereavement have the potential to lead to misdiagnoses within a healthcare system bound overwhelmingly to the biomedical model (Bhugra et al., 2021; Bolton & Gillett, 2019; Summerfield, 2012). In this vein, psychiatrists and systems must take care not to pathologise natural or culturally-sensitive reactions to complex circumstances (Blackwell, 2005 cited in Persaud et al., 2019). Greater investment in developing “culturally capable” (Edge & Bhugra, 2016, p. 24) care is required to enable individuals and their networks greater agency over their ongoing care and recovery (Abdi et al., 2021; Edge & Bhugra, 2016; Wei et al., 2021).

### Culture and Recovery

The link between socio-cultural context and cognitions around recovery has been established (Kalathil et al., 2011; Kirmayer & Jarvis, 2019; Knifton, 2012), yet few studies have specifically investigated CALD or minority communities’ experiences in this domain (Brijnath, 2015; Kalathil et al., 2011; Matsuoka, 2015; Southside Partnership Fanon, 2008, as cited in Slade et al., 2017; Tang, 2019; Virdee et al., 2017). People of different cultural backgrounds may approach communication in ways other than the linear notions common to native English-language speakers, such as a gradually building “roundabout style” or by using less powerful language (Meadows, 2017, p. 21). Sector and lived experience leaders in Australia have emphasised the importance of shifting to speaking about *wellbeing* rather than *mental health* due to varying explanatory models of health, and CALD communities not identifying with the Australian medical model (Katsifis et al., 2017).

The constructs of *relational recovery*, where people are seen as inseparable from their supportive social contexts, and *family recovery*, where individuals’ interpersonal or familial roles are considered, may be well suited to people in cultures with more collectivist values (Glynn et al., 2006; O’Hagan et al., 2012; Price-Robertson et al., 2016; Tse & Ng, 2014). However, given the processes of globalisation, the notion of pure culture arguably no longer exists (Tse & Ng, 2014). Milasan (De Montfort University, 2021) was also critical of contemporary recovery models due to their lack of cultural sensitivity.

Despite some service users demanding greater intersectionally-informed care, services remain largely oriented around individualistic conceptualisations of recovery which are not always culturally sensitive (Morrow & Weisser, 2012; Price-Robertson et al., 2016; Summerfield, 2004). Slade et al. (2014) highlighted that despite the CHIME (Connectedness, Hope and optimism, Identity, Meaning and purpose, Empowerment) framework emerging from a systematic review of the concept of personal recovery (Slade et al., 2012), the notion of recovery and related assumptions may still be “monocultural” (p.17). Others question if the framework imposes an overly optimistic understanding of recovery that could result in a sense of shame if one’s experiences were not congruent with the framework (Stuart et al., 2016). A scoping review by van Weeghel et al. (2019) has since recommended that the elements of ‘difficulties and trauma’ be added to and supplement the existing CHIME framework to address some of these perceived omissions. In a recent study (Llewellyn-Beardsley et al., 2020), individuals from under-researched (including ethnic minority) populations in England were interviewed to expand upon a typology of recovery narratives. The study expanded the conceptual framework in many ways, including the importance and value of non-verbal recovery narratives. While the *Finding our way* (Movie-ment, 2014*)* films included recorded narratives, they allowed for other modes of expression, and for multiple understandings of mental health and recovery (Slattery et al., 2020; Stickley et al., 2018).

### The Australian Context

A recent joint recommendation report by the Ethnic Communities’ Council of Victoria and Victorian Transcultural Mental Health, two bodies who helped fund the initial *Finding our way* study (Movie-ment, 2014), recognised that the state of Victoria is a “society that is defined by diversity” (Plowman & Izzo, 2021, p. 3). Given this, the report condemned the Eurocentric culture of the state and national healthcare systems in Australia, suggesting it alienates those of migrant and refugee backgrounds who understand diverse models of health and wellbeing, and puts people at risk of negative health outcomes. On a national scale, immigrant and refugee populations are consistently left out of mental health research and evaluation, the impact of which is “large and persisting gaps in knowledge” about the mental health of these communities (Minas et al., 2013, p. 20). One systematic review (Enticott et al., 2017) called attention to strategies for incorporating refugee and asylum seeker groups in health-aligned research, in the hope of including greater representative samples of these populations in the future. However, practitioners, researchers, and policymakers need to remain conscious of the structural inequities and relationships which may unintentionally create positions of disempowerment and the potential of recovery for individuals (Bracken et al., 2021; Brijnath, 2015). Furthermore, while the involvement of service users in mental health research through methods of, for example, co-production or co-design, has been increasing, individuals of CALD backgrounds are still grossly underrepresented in these groups (Diocera & Colucci, in press; Synergi Collaborative Centre, 2019).

### Recovery Narratives and Visual Research Methods

The underrepresentation of CALD communities in research was also observed by the coordinators of the *Finding our way* project and contributed to the project’s initiation around the meanings of recovery in CALD communities (Colucci & McDonough, 2020). In the years since the initial study (Colucci & McDonough, 2020), various forms of participatory research have increasingly been used to attain lived experience perspectives and in particular, with those from underrepresented communities (Abdi et al., 2021). A participatory mental health research approach with refugee communities is useful given the particular relationship between culture and the experience of psychological distress encountered amongst CALD persons (Hwang et al., 2008). Applications of visual methodologies have offered unique ways to engage people from marginalised communities, and to challenge norms where these may impede recovery or the mental health of members of these communities (Colucci & Bhui, 2015; Diocera & Colucci, in press). There has also been an increase over this period in arts-based and participatory studies focusing specifically on mental health recovery (Casey & Webb, 2019; Doroud et al., 2021; LaMarre & Rice, 2016; Milasan et al., 2020; Petros et al., 2016; Vansteenkiste et al., 2021; Whitley et al., 2021). Scholars suggest these approaches draw on the narrative properties of creating art, and that the process of art-making itself in various forms can be both meaningful to participants, and help to illuminate stories of lived experience in more complex and multidimensional ways than possible through word-based narratives alone (Casey & Webb, 2019; Gubrium et al., 2014).

The purpose and contexts for which recovery narratives are constructed need also to be considered, due to the risk that certain narratives may be produced under coercive conditions, subjected to censorship, or be required to conform to a cultural script (Kaiser et al., 2020). Other authors have cautioned that certain recovery narratives are becoming dominant over others that do not conform to societal ideals (Morgan et al., 2016; Nurser et al., 2018; Woods et al., 2019). For refugee communities who are often “affected by complicated legacies and current experiences of violence, deprivation, and discrimination”, recovery should aim to ameliorate past violations and prevent future violations (Jacobson & Farah, 2012, p. 335). Recovery must also include the notion of healing, work to address trauma, and reduce the “impact of isolation brought about by cultural and language barriers” (Ida, 2007, p. 49). Intriguingly, some scholars (Summerfield, 2004) question the notion not only of recovery, but also constructs of *processing*, *healing,* or *closure*, as they separate the mental from the material world which the scholars suggest leaves them susceptible to being instrumentalised.

The digital storytelling (DST) project that led to the production of the *Finding our way* films did not seek “to promote any pre-determined issues or perspectives” (Colucci & McDonough, 2020, p. 212). Further, the current study agrees with scholars who cautioned against appropriating powerful individual narratives into “professionally derived conceptual [recovery] frameworks” (Harper & Speed, 2012, p. 21) and sought to fully situate the individuals’ unique recovery narratives within their social and cultural contexts. To counter the homogenisation of lived experience recovery narratives (Woods et al., 2019), the current study sought to expand both understandings and the literature on experiences of recovery in people from under-represented, non-dominant cultural groups and their narratives. The study investigated the nine publicly accessible visual narratives about mental health recovery resulting from this project using a thematic orientation and method informed by the social justice principles of equity, access, participation, and harmony (Crethar et al., 2008; Lyons et al., 2013).

## Methods

### Aim

The aim of this research was to explore, in depth, the stories and experiences of recovery shared by the participants in order to advance understanding of the phenomenon under investigation. The Thematic Analysis approach adopted (Braun & Clarke, 2006, 2020) was inductively driven to respond to the research question: *How do individuals of culturally and linguistically diverse (CALD) backgrounds understand and express their lived experience of mental health recovery within the Australian context?* The contentious connotations of the CALD acronym, its currency, transparency, as well as its breadth and intersectional applicability are acknowledged (Mousaferiadis, n.d.; Sawrikar & Katz, 2009). The term was used to situate the research best in related literature and sought to avoid reinforcing discriminatory language.

### Study Design

The current study involved secondary analysis of data collected in Melbourne, Australia by Colucci and McDonough in 2014 in a community engagement project called *Finding our way,* which was conducted as part of the Mental Health in Multicultural Australia (MHiMA) Federal Government initiative (Colucci & McDonough, 2020). The project invited participants “of immigrant and refugee background[s] with a lived experience of mental health or emotional issues” to partake in a four-day digital storytelling workshop (Colucci & McDonough, 2020, p. 206). With the use of digital storytelling (DST), a visual methodology increasingly used in mental health research (De Vecchi et al., 2016; LaMarre & Rice, 2016), participants created first-person narratives set within an image context to explore personally relevant aspects of mental ill-health and recovery. Ten participants were assisted by their support persons, trained facilitators, and project coordinators to construct personal stories visually. Each participant had full control over their self-representation, organised their ideas, and constructed how story elements came together into the final film form (Colucci & McDonough, 2020). Ten short films were created by the participants of between approximately two minutes and 45 seconds, and five minutes in duration. The nine that are accessible to view online in 2021 form the dataset of the current study.

### Participant Recruitment

The participant recruitment process for the initial *Finding our way* participatory research project can be found in Colucci and McDonough (2020) and McDonough and Colucci (2021).

### Participant Information and Consent

All stories that were made publicly available were produced with the written consent of each respective storyteller, each of whom retains the copyright to their own content (McDonough & Colucci, 2021). Participants were invited to sign a release form authorising the digital storytelling agency and project partners to screen the content, which allowed for them to contact the agency and end the agreement at any time. This consent gave permission to screen the stories for promotional and educational purposes, and to make them available on agency websites (McDonough & Colucci, 2021).

### Data Analysis Utilised in the Current Study

Thematic Analysis (TA) is a method aimed at apprehending themes of lived experience of participants (Braun & Clarke, 2006; 2020; Crowe et al., 2015) and was utilised to analyse the data from the *Finding our way* transcript material. Thematic analysis is an embedded, rigorous but flexible tool that allows researchers to synthesise and interpret patterns in data (Braun et al., 2019). A researcher, from their own analytical position, adds meaning to those patterns to tell a story, grounded in the data and “in conversation with other research” (Braun et al., 2019, p. 12). Thematic analysis is commonly used in lived experience and mental health research (for example Braun et al., 2014; Crowe et al., 2015; Graham & Clarke, 2021). In seeking to unearth salient themes in the dataset (Attride-Stirling, 2001), the primary researcher took the stance that meaning is socially constructed (Berger & Luckmann, 1966). In taking such a critical realist perspective (Lyons et al., 2013), the researcher’s personal identity and cultural influences undoubtedly influenced the way she constructed meaning and perceived patterns in the data. However, she was careful to conduct this process reflexively, phenomenologically, and with sincerity (Tracy, 2010) as further observed below.

### Conducting Thematic Analysis

Thematic analysis was conducted in accordance with the steps laid out by Braun and Clarke (2006). The narratives of each of the nine *Finding our way* films created by participants in the MHiMA digital storytelling project were initially transcribed. The primary researcher coded the data inclusively, which involved giving each data item equal attention through the process, and allowed for data to be coded into multiple potential sub-themes where appropriate (Braun & Clarke, 2006). The researcher then searched for patterns of meaning across the dataset related to mental health recovery in CALD communities and endeavoured to hold space for any other developing themes of relevance to the research question (Braun & Clarke, 2006). Through the collation of relevant coded data, substantial sub-themes, and then fully developed themes were created. A theme was conceptualised as uniting data that captures the essence or meaning of experiences, of one or more individuals in multiple instances (Braun & Clarke, 2006; DeSantis & Ugarriza, 2000). The results of the preliminary thematic analysis were then analysed by supervisor Dr. Colucci, and subsequent iterations of the analysis were conducted following discussions where differences existed between both parties until consensus was reached. Through this interpretive, reflexive process, the themes began to tell a story about the dataset as a whole (Braun & Clarke, 2020).

Access to the participants’ films was located via the *Finding our way* project page on the Movie-ment website (https://movie-ment.org/findingourway/).

### Reflexivity

The primary researcher took care to practice reflexivity throughout the analysis process, seeing her subjectivity as an “analytic resource” (Braun & Clarke, 2020, p. 3). Self-reflexivity was of critical importance in this study where an intersectional approach was used to explore complex experiences of the Other (Talwar, 2010). The primary researcher endeavoured to examine personal biases that arose through her own experiences of the Australian healthcare system and was careful to mitigate these as such. They endeavoured to bracket out their own values and lived experience to read the contributions of participants in a phenomenological manner, holding true to the intentions of each person. They also considered institutional and societal biases that may have contributed to the analysis process. They reflected on how it felt to analyse personal stories of individuals they had not met, and how being further removed from the participants might impact upon this study or any findings (Lincoln, 1995).

### Secondary Qualitative Data Analysis: Rigour, Value, and Ethics

The current study was written under the supervision of one of the initiators and authors of the *Finding our way* participatory research project, Dr. Colucci. This added rigour to the current study through her “understanding of proximate contexts” (Morrow et al., 2014) which reduces the potential of misappropriated interpretation of data and misrepresentation of participants and fills any possible gaps in lack of first-hand knowledge (Ruggiano & Perry, 2019; Thorne, 1994). Finally, as the participants gave literal voice to their own stories which formed the content analysed in the current study, chances of misrepresentation were reduced.

## Results

In this section, the findings resulting from the analysis of the data from the nine participant video transcripts is presented, along with a demonstration of how each of the five, distinct final themes related to the key study question were conceptualised through an interactive and iterative process (Braun & Clarke, 2020).

**Fig. 1:**
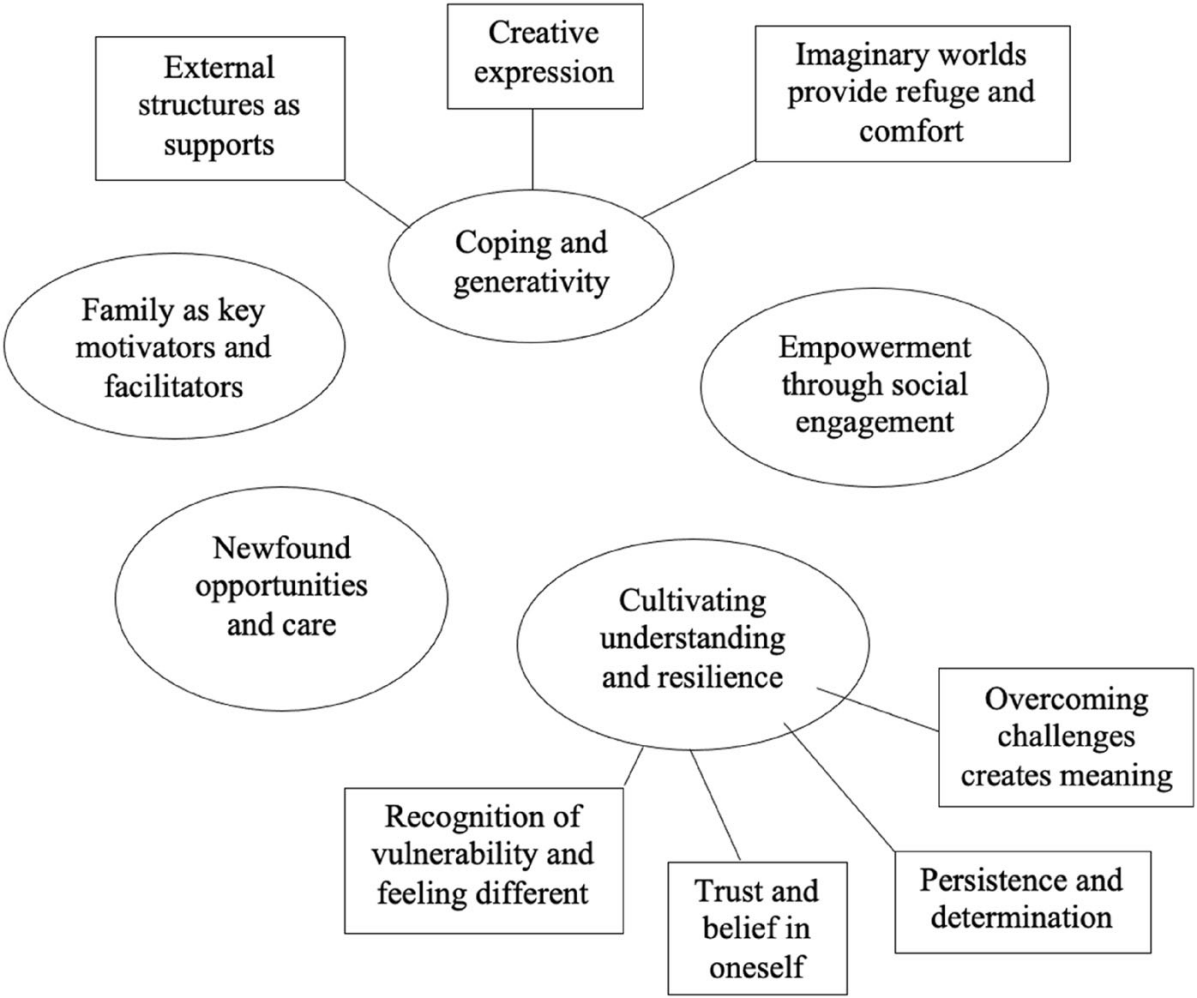
Final thematic map, showing five final themes and seven sub-themes

Two themes were created directly from the data without informing sub-themes. These were: *Newfound opportunities and care* and *Family as key motivators and facilitators*. The third theme, *Coping and generativity* encompassed the sub-themes *External structures as support*, *Creative expression*, and *Imaginary worlds provide refuge and comfort*. The fourth theme *Cultivating self-understanding and resilience* was created from the sub-themes *Recognition of vulnerability and feeling different*, *Trust and belief in oneself*, *Persistence and determination*, and *Overcoming challenges creates meaning*. The fifth theme *Empowerment through social engagement* resulted from the analysis process particular to the data of a single participant. The themes are shown in the thematic map below (Fig. 1). Table 1 shows examples of the thematic reduction where short oral transcripts from the digital stories are coded as themes and subthemes. The analysis aligns examples of participants’ experiences to the five main themes that emerged. A discussion of each final theme then follows.

**Table 1:** Thematic reduction

Themes	Sub-themes	Participant	Supporting quote
Newfound opportunities and care		Doe Doh	I have opportunities to imagine and plan my future now
Family as key motivators and facilitators		Maria	Children are important to me and I believe they keep me young
		Chandima	I finally found my lifetime partner. I got married and had four beautiful children, three boys and a one girl. I adore them dearly
Coping and generativity	External structures as support	Akeemi	We are finding real people to help us now
	Creative expression	Linh	I was an inpatient in hospital for three years under the care of doctors, nurses, and medication
		Tuan	I identify colours as a spectrum of life embellished with my understanding. When I put things together, I feel enlightened about the goodness around me. That joyful moment, I can't put into words
	Imaginary worlds provide refuge and comfort	Akeemi	You created dream people to hide behind
Cultivating self-understanding and resilience	Recognition of vulnerability and feeling different	Nevena	We left because the authorities wouldn't allow us to stay longer. We were refugees
	Trust and belief in oneself	Maria	[I] am now a more empowered and resilient person
	Persistence and determination	James	Mantras are a colourful, powerful device in my graphic novels. I use them to take back my power from the negative. For two hours every day I go into the workshop of the mind and practice. Like playing the scales of the piano
	Overcoming challenges creates meaning	Akeemi	We will jump in a sailboat, with a camera, and capture our colours and our black. We will try to see the colours of life again today and/on the next day, and the next
Empowerment through social engagement		Kim	It makes me very happy to share food that I have grown and cooked myself

### Theme: Newfound Opportunities and Care

Positive experiences within the Australian healthcare system and in engagement with the Australian people resonated strongly throughout the narratives of two participants, Linh and Doe Doh. Linh, who migrated to Australia in 1990, stated: *“The government, through the Human Services, the carers, the social workers as well as the psychiatrist have helped me.” (99–100).*

Gratitude for the practical care she and her family received from the Australian community was expressed and how this led to feelings of belonging: *“The Australian people have been wonderful; some have been caring for us by giving us accommodation to get on with life. ‘I still call Australia home’ just like Peter Allen in his song.” (108–110).*

This acknowledgment and reference to the iconic 1980 hit song by Peter Allen “I Still Call Australia Home” suggests Linh’s personal process of identifying with Australia and becoming comfortable with having a place in the country. Doe Doh, who was granted refugee status by the UNHCR, similarly referred to new opportunities that emerged for him, such as a sense of human rights, in Australia:

*Coming to Australia gave me my freedom, freedom to do what I want to do, and to be who I want to be. The opportunity to change my life. Having rights in Australia and opportunities showed me that [if] I can make it here then I can make it anywhere. (60-63)*

Both participants arrived in Australia as young persons, and as a result of spending some of their formative years in their new home country, and under more favourable and caring conditions, were able to develop a sense of confidence and belonging. The data from Doh Doe suggested his experiences prior to coming to Australia stood in great contrast to the care and opportunities offered in his new home.

### Theme: Family As Key Motivators and Facilitators

In contrast to care and opportunities emerging from the Australian services system and the people who work there, Chandima drew attention to a more personal dimension of support in the family. Chandima came to Australia as an adult, leaving her family of birth behind during the civil war in Sri Lanka. She highlighted her love for family, rather than directly for the Australian service context in providing the grounding that was helpful for recovery. Chandima stated: *“My anchor was my husband, and my children and my family overseas.” (129).*

Maria, of Greek heritage, born in Australia, discussed how her relationship with her family has changed over the course of her life. She described a cultural upbringing that *“conditioned me as a female to care for my parents [a role which] over the years has inhibited my growth”. (178–179)* However, Maria explained that she did learn *“to put myself first” (179–180)* and stated: *“For me, family is key to wellness. My family and extended family are a very important part of my life.” (196–197).* For both Maria and Chandima, family was stated as central to their positive experiences of daily living and thus, to their recoveries.

### Theme: Coping and Generativity

Various forms and methods of coping and living with mental illness and in recovery were discussed by almost all participants. Some participants told of utilising external structures and supports, periods of hospitalisation, or particular technologies, while others turned to creative endeavours or imaginary worlds for refuge and comfort in their coping strategies. Arguably, some of these coping strategies generated new personal capacities and growth. This is evidenced in James’ description of his Lightwarrior Superhero’s journey: *“…the experiences of the Psychiatric Superhero and the strategies he employs to triumph over evil are drawn from the knowledge and practices I have developed in my daily practice of managing schizophrenia over many years.” (145–148).*

While both service systems of care and family are also directly related to how participants coped in their lives, the qualities represented in this theme are distinctive in that these supportive structures relied less on a personal human interface.

Some participants spoke of external structures as supports. Chandima’s faith served as a key support structure. *“Faith is the reason why even in pain, I smile. In confusion, I understand. In betrayal, I trust. In fear, I continue to fight.” (111–112).*

She described trying her best to carry out responsibilities but that a *“darkness… got hold of me” (122–123)* and she found herself drifting away from God.

*True peace does not come as a result of eliminating sorrows and disappointments, it comes as a result of one thing, and that is an intimate relationship with the Lord Jesus Christ. To me, he is where anxiety ends, and peace begins. Faith is the significant element in my recovery journey. I’m thanking God for my family and friends who prayed for my recovery. (125–129)*

In contrast, Tuan referred to the practical support on a day-to-day level, of using a GPS or phone to seek help when he was lost while out in the community. *“Sometimes I find things I can use in my art, sometimes I get lost, and I find [sic] difficult getting back to my house, so I use GPS or call someone familiar.” (83–85*).

Four participants expressed methods of coping through creative expression. Kim utilised two avenues to channel her emotions in a range of ordinary everyday contexts.

*I started to make origami fish. I used red pockets to make fish to calm myself down. I started making fish whenever I could. In the house there were fish everywhere, all over my table, chairs and even the floor… In 2003, I learned to be a laughter leader. At that time, I found out this is the only exercise I can do at anytime, anywhere. Even in my situation, I could still laugh. (160–162, 165–167)*

Tuan similarly adopted a creative imaginary mode of coping:

*I’m colours. I love my colours. I see life around me as colours and shapes… I swing between different planes, different dimensions. Colours are a pathway to reality for me. Counting buttons or breads help me to feel safe, so I don’t feel like hiding under the doona or having so many showers in a day. (70–71, 87–90)*

Maria’s creativity was a dominant thread throughout her narrative of recovery, as she spoke of her career as an artist. Emerging at a time where she felt deeply inhibited as a person in a culturally and domestically challenging period, Maria developed an arts practice that helped her cope in life and realise new potential:

*In year 12, I chose the medium of ceramics…my art teacher who persuaded me to apply for a degree in Fine Arts... I studied and graduated as a formal visual artist ... (and) ... I exhibited and curated my work around Melbourne galleries. I also completed a postgraduate diploma in Arts Curatorship and Museum Management. And I developed my own decoupage technique (and) I became represented by* archsign *style gallery in Victoria. (180–186)*

Creative expression had been a primary method of coping for James, and as a way to understand his experiences of some symptoms of schizophrenia and the impacts of his drug use. James converted this coping strategy into an enduring focus on and creation of a graphic novel featuring his invention of the Light Warrior Psychiatric Superhero. *“I’m endeavouring to visually display this archetypal journey from the state of being cursed to one of liberation, and through the madman’s trials and tribulations, illustrate how to become his own healer and healer redeemer.” (143–145).*

Using the powers of imagination Akeemi also discussed her creation of an alternate fantasy world she inhabited, populated with imaginary people to help her cope as a child after her mother left the family.

*Someone to clothe and bathe you, feed you and rock you to sleep. Someone to chase away bad dreams. Someone to play with. Sometimes they felt more real than the real people. They kept you feeling safe and cared for. That was okay. As you grew older your needs changed, so you created different people to support you. (33–36).*

The diverse avenues and forms through which participants expressed their coping speak to the myriad ways in which both implicit and explicit structural, spiritual, creative, and self-protective measures can benefit those living with mental ill-health and in recovery. The emergence of, and role played by, creative activity in recovery is also emphasised in these narratives. While some participants emphasised a key or favoured coping strategy, others highlighted multiple ways of managing their needs. We are privy to the ways in which Kim and Chandima’s experiences of expression and coping have changed across the years. We also learned that despite other supports coming and going, Chandima’s faith had been a constant across her life. These examples demonstrate the varied ways and sometime shifts participants employed to cope and access support over time.

### Theme: Cultivating Self-Understanding and Resilience

All nine participants spoke of ways of cultivating resilience through an understanding of themselves and the world around them. For many, a sense of being ‘other’ or different from those around them left them feeling vulnerable or ill-at-ease. However, at the same time, many participants also expressed deep compassion for their present and past selves, recognising what led to their ill health and the ongoing anxieties they experienced.

Akeemi adopted a kind of self-talk addressing her “*little self*” *(28)* directly, and in a self-nurturing gesture offered this *“little self” (28)* apologies for the pain she experienced.

*You were a blue child, always sick, always alone. In pain, fear, hunger. In perpetual darkness. Mum had to leave with your siblings. You must have felt unwanted, uncared for*… *You must have been lonely, being surrounded by more and more people, who did not know who you really were. I’m sorry. (28–30, 43–44)*

Both Nevena and Chandima left families and lives behind in war-torn countries, and came to Australia as refugees, resulting in experiences of culture shock and struggles to survive, as Chandima shared: *“[I] arrived in Australia, I was trying to survive. It was a cultural shock.” (114–115)*. Nevena also reflected:

*It was cultural shock when I arrived in Australia. I had to retrain my thinking about everything. Everything my culture had expected of me was accomplished. Was born, went through education, found a job, got married, have had children. But that was the culture of the land that I came from. I needed to move in a science of mind. Learning how to live the life of my eventual new homeland. (10–14)*

Both women expressed the complexities of navigating a new environment with very little support, and the resilience required to manage fundamental differences in everyday life to those they had left behind. Nevena, who fled her homeland with her three sons, and also lost her husband, had repeatedly been tasked with finding a new home that would accept them as residents for the long term:

*War came to my home in Sarajevo. I had to leave with three sons: four, eight, 10. My husband stayed behind. I fled to Belgrade. Didn’t know anyone there, but it was the only place to go… Only a month later, my husband was killed by a neighbour. His childhood friend called me from Sarajevo to tell me. I was 38 years old. I was frightened, but I had to move forward for my children. (1–6)*

Doe Doh, also a refugee to Australia, grew up as a Karen boy living in the Thailand-Burma border:

*As Karen refugees we had no rights, no freedom, no safety, no opportunities, no hope, or purpose for the future… Because of the war and poverty, other reasons as a child, you were not treated well. They did not teach us how to become someone. I grew up with ‘negatives’. My culture was restrictive and conservative. I could not express myself freely and there were strong expectations. (51–52, 56–58)*

Doe Doh’s experience of life before Australia contrasted with both Nevena and Chandima’s experiences of having a home and established sense of self in another country. Interestingly, the analysis suggested that for both women, Australia presented consistent challenges of adaptation, relearning, and rebuilding from scratch. The way Doe Doh spoke of his life in Australia expressed challenge but was imbued with hope for building himself into a person he did not even know he had the potential to be.

*It [having rights and opportunities in Australia] helped me to build my confidence and to realise no one can stop me. I didn’t know anything about life before. My experiences here, as well as developing and maturing over the past five years has helped me change, learn, and grow stronger… I just need to take my time and focus and keep the belief that I can be someone. (63–66,69)*

Chandima and Nevena also expressed how overcoming the challenges of coming to live in Australia produced opportunities for growth and making meaning out of adversity. As Chandima stated, *“Challenges are what make life interesting and overcoming them is what makes life meaningful. I have had many disappointments, but I learned from all of my experiences.” (130–131)* Similarly, Nevena revealed how, *“Finally, everything popped, and I was able to understand, accept and respect. It helped me to connect and appreciate the good and bad in life.” (23–25).*

Yet, Nevena also spoke of the determination it takes to persevere with living, amidst mental and physical pain, *“I still reflect and struggle with these [painful] feelings today. There are so many layers inside and you are never free. However, I’m a proud being and grateful of my transformation and the life I have.” (25–27).*

Akeemi shared a similar sentiment with her younger self: *“It’s okay little one. Life does not need to be perfect. Good enough is okay. Let the black mix with the colours and know that the picture looks even prettier that way.” (47–49).*

James strongly expressed how overcoming his inner demons became meaningful as he intertwined and externalised his experiences into his graphic novel.

*I decided a long time ago that I was going to outrun the voices. I would drown them out with my own inner voice. My core mantra “I take back my infinite power from the negative, even here, even now”, will be woven into the fabric of the tale. (153–155)*

Linh’s persistence and determination brought her to a place where she was more able to care for herself, and able to be independent in certain contexts, *“Now, I can go to school twice a week to learn more of what I want to know. By joining the group with daily helpful activities, I can now cook, wash, and clean up the home.” (101–103).*

In differing ways, each of the participants spoke of their own growth in self-understanding and capacity to manage throughout their recovery except Tuan, whose clarity in his sense of otherness and contentment was rather unique:

*People don’t make sense to me, no one makes sense to me… I question myself. I ask am I different to other people? Many moons ago I would have been contributing to the community and living the concept of average life*… *A lot of professionals talk about healing and it’s another confusing thing. I’m not keen about healing, it’s more of a resolution. (71, 86–87, 90–91)*

In this way, the data corroborate how no uniform terminology or linear recovery curve is applicable to all participants.

The participants each expressed an awareness of their personal capacities to cope with and manage their health and recoveries. The challenges faced were multiple and far-ranging, from the practical tasks related to becoming independent and self-satisfaction, to the difficulties of cultural adaptation and traumatic migration histories. The data suggest that coping and generativity are ongoing processes that involve developing certain new skills and reaching milestones but may equally be a process of managing daily symptoms through medication, self-discipline, or interpersonal support.

### Theme: Empowerment Through Social Engagement

For Kim, interpersonal engagements outside of her close circle of family and friends were identified as key to her experience of recovery. The attraction to participating in social interactions and building relationships with others was a consistent theme. She reflected that when making origami fish, a creative method of coping and self-soothing, at Christmas: *“I took my fish and jumped on the city trams. I gave my fish to the tram drivers and passengers and said ‘Merry Christmas’. They were all surprised. Then I also began teaching my neighbours to make fish.” (163–165).*

Kim discussed a further expressive activity, that of laughing. She drew attention to the manner in which she contributed to the wellbeing of others through her role as a ‘laughter leader’ engaged with the community. *“I still go to different communities to bring my laugh to both old and young.” (168).*

Finally, Kim discussed the value of cooking for others as part of her recovery:

*I also cook for different community events at the Chinese Buddhist temple. People say my K. L. C. (Kim Ling Chicken) is better than K. F. C. I love to cook. Every time I see people leaving an empty plate, it gives me great pleasure and a big reward. (170–173)*

Kim’s narrative placed emphasis on how learning new skills, but particularly being able to share them in engagement with others, has been an empowering factor in her recovery.

## Discussion

The current study explored first person lived experience stories of mental health recovery from nine persons of CALD backgrounds in the Australian context. This study involved conducting reflexive thematic analysis (Braun & Clarke, 2006; 2020) of participatory digital storytelling research conducted by Colucci and McDonough. Nine narratives were analysed, from which five final themes were developed. The themes, like the CHIME framework (Slade et al., 2012) itself, illuminate and exemplify future-focused stories of hope and meaning. While the supplementary dimensions of ‘difficulties and trauma’ suggested by van Weegal et al. (2019) are distinctive in focusing on less positive aspects related to recovery, they add richness and speak to the lived experience of many participants in this study. As such, the findings of this study draw correlations to the CHIME conceptual framework for personal recovery (Slade et al. 2012), including van Weegal and colleagues’ supplementary dimensions. The following discussion braids these elements into a narrative aimed at advancing understanding of lived experiences of the participants of the *Finding our way* project. The discussion offers a more expansive version of the CHIME factors beginning with findings related to Connectedness, Identity, and Empowerment (Slade et al., 2012; van Weeghel et al., 2019). The other key CHIME factors of Hope and optimism and Meaning and purpose feature throughout the discussion.

The *Finding our way* stories feature the CHIME elements of Hope and optimism and Meaning and purpose in much the same way that the CHIME literature describes (Slade et al., 2012; van Weeghel et al., 2019). The elements Connectedness, Identity, and Empowerment were also important to these recovery stories. Importantly, when it came to exploring their perspectives on mental health recovery, the storytellers contextualised these elements in particular ways; that is, their stories expressed Connectedness *and* coping, Identity *and* gratitude, and Empowerment *and* resilience. The following discussion considers how these three more expansive themes of mental health recovery resonate with having a recent or family story of migration, or being a refugee.

### Connectedness and Coping

Van Weeghel and collaborators (2019) drew attention to the challenges of trauma, loss, and bereavement during the recovery journey, and to the creation of meaning for people with mental illness. This was salient to the experiences of some participants in the current study, including the impacts of post-traumatic stress resulting from forced migration. Prior to fleeing their war-ravaged homelands two participants, Nevena and Chandima, had led full lives with families and fulfilling relationships. Nevena fled with her children, while Chandima established her own family once in Australia. However, both women were forced to leave behind anchors in their countries of origin and experienced severe destabilisation upon migration, and both expressed guilt for leaving or surviving what some others did not (Bhugra et al., 2010; Puvimanasinghe et al., 2014). As such, their senses of personhood post-forced migration should be considered “in the context of developing culture bereavement, culture conflict and culture shock” (Bhugra et al., 2010, p. 144), or what Berry (2018) referred to as, acculturative stress. Greater acculturative stress and cognitive dissonance related to attempts to adapt to the values and expectations of one’s new environment can lead to cultural bereavement and an experience of internal culture conflict (Bulik & Colucci, 2019; Bhugra et al., 2021). Nevena offered a practical example of this when she told of learning to walk on the left-hand side of the street. For her, this involved a repeated internalisation of the cultural norms and the realisation that when bumping into people, she was in the wrong. Furthermore, bereavement constructs common to post-colonial nations such as Australia have limited value when explaining what diverse expressions of grief are in other cultures (Bhugra & Becker, 2005). The extent to which one feels they have retained a locus of control of the events leading to their migration (such as whether they feel responsible) also influences the bereavement process (Bhugra et al., 2010) and overall wellbeing (Alim et al., 2021).

Both Chandima and Nevena gave voice to the difficulties of mothering during extreme stress and the lack of support from social networks and their families of origin. This was referred to by Edge and Bhugra (2016) who underscored the immense stress of a woman’s responsibility in maintaining a family without extended family support, as well as how discrepancies between cultural expectations around gender and women meeting their own needs can lead to dis-order (Bhugra et al., 2010). This is particularly noteworthy in women of ethnic minority background for whom cultural racism and misogyny can further increase mental vulnerability (Bhugra et al., 2010).

### Identity and Gratitude

Complexities related to identity formation (the “I” in the CHIME framework) and change were present for many of the participants and are reflected in the themes of *Newfound opportunities and care* and *Cultivating understanding and resilience*. Maria’s experience of living with her family of origin was a source of difficulty during adolescence, where her personal values as an Australian of Greek heritage were incongruent with the roles expected of her in traditional Greek culture. Experiences of such culture conflict are common in people with “hyphenated identities” (Edge & Bhugra, 2016, p. 16), when there are discrepancies between a person’s cultural values and those of their family or larger communities (Bhugra et al., 2010; Edge & Bhugra, 2016; Ziaian et al., 2021). Edge and Bhugra (2016) stated that widespread adoption of such “ethno-cultural signifiers” (e.g. Greek-Australian) in certain societies including Australia, the United Kingdom, and the United States, have the potential to impact a person’s mental health due to tensions around belonging and belief. A study by Ziaian et al. (2021) on the experiences of youth from refugee backgrounds and their significant family members, included the voice of a young woman born in Iraq. The participant considered herself “half-half” (p. 133), a shared sentiment among participants who found some resolution in a dual identity. Furthermore, Berry and Hou (2019) found that having a sense of belonging to multiple groups beyond national and heritage categories, or having “multiple identities” (p. 140), can also have psychological benefits, including providing opportunities to develop resilience (Ysseldyk et al., 2013). Davidson (2019) argued that personal recovery is overwhelmingly about “the quality of a person’s sense of identity and belonging to a community” (p. 1079). Thus, both affective and cognitive dissonances related to attempts to assimilate values of two cultures (Inman et al., 2001) and perhaps a developing emerging identity, would likely influence one’s ability to work towards recovery.

A sense of profound gratitude for the opportunity to resettle in Australia was present in the lived experience narratives of the present study and was also reflected in Puvimanasinghe et al. (2014) who explored the experience of Burundian and Sierra Leonean refugees’ transition to settlement in Australia. Their expressions of appreciation for safety, the support of governmental services, and new and greater opportunities after resettlement echoed those of participants Doe Doh and Linh, who came to Australia during their youth. Ziaian et al. (2021) studied the experiences of youth of refugee backgrounds and their significant family members and found similar expressions of gratitude for the “opportunities, safety and support available in Australia” (p. 129), to achieve one’s dreams, as well as a desire to “give back” (p. 129) and contribute in return to the new home country. Other research (Piat et al., 2017) suggested both receiving and providing support indicates a broadening of identity dimensions. However, it is pertinent that participants in these studies (Puvimanasinghe et al., 2014; Ziaian et al., 2021) as well as others (Fozdar & Hartley, 2013) such as Kale et al. (2018) in the New Zealand context, and including this study, focused largely on the feelings of gratitude for civic provisions, such as services and healthcare, rather than feelings of welcome from the wider Australian community.

### Empowerment and Resilience

Emergent feelings around a sense of *Empowerment through social engagement* and *Cultivating self-understanding and resilience* found in this study correlates with the “E” in the CHIME framework (Slade et al., 2012) and resonated strongly with other recent participatory research undertaken with people with migration histories and mental health challenges. A recent, small study (Jannesari et al., 2019) highlighted the significance of asylum seekers’ identity loss after periods of stressful transition into a new cultural context. The authors found that an absence of appropriate spaces for healing from the huge upheaval and risks involved in the flight to refuge, led interviewees to cope “by forgetting, locking experiences and emotions away” (p. 9). The voice of one participant of that study, Shelly, resonated strongly with that of Nevena in the current study. Both women expressed a bubbling up and suppression of emotions that had negative impacts on their overall health and wellbeing.

As emphasised earlier in this discussion, access to culturally responsive, trauma-informed care and diverse supports is critical to overall wellbeing and recovery (Abdi et al., 2021; Edge & Bhugra, 2016; Minas, 2018; Plowman & Izzo, 2021; White et al., 2016). Jannesari and colleagues (2019) surmised that being involved in collaborative opportunities to share their experiences may have been therapeutically beneficial to participants through the creation of “a trusting environment in which interviews could recount and reframe traumatic experiences” (p. 9). In the follow up reflective study (McDonough & Colucci, 2021) with participants of the *Finding our way* project, similar sentiments around self-expression and a sense of being heard arose. Additional participatory arts research with refugees and survivors of torture in the UK (Rose et al., 2018) utilised Herman’s (1992) three-stage model of recovery from trauma, which identified safety, remembrance and mourning, and reconnection as key non-linear stages of the recovery process. The researchers observed the creative participatory environment to facilitate connection to a changed identity through empowerment, and the integration of trauma into participants’ autobiographical identity. This is based on Lamb’s (2017) goals of therapeutic care with trauma survivors, and is also apparent in many of the narratives of participants in the *Finding our way* study.

While the aims of these studies were not explicitly therapeutic, findings suggest participatory projects do have the potential to empower participants (Casey & Webb, 2019) to talk about their recoveries (Anderson Clarke & Warner, 2016) and “lead to paradigm shifts that can provide a roadmap to effectively address disparities in mental health” (Abdi et al., 2021, p. 1). Such ideas are in line with experience-centred research addressing illness narratives founded on the understanding that the process of telling one’s own story and expression of deep feeling can be empowering, have healing effects, and can help with sense-making and strengthening of identity (Lawrence et al., 2021).

The theme *Cultivating self-understanding and resilience* that developed through the analysis process is particularly salient as it encompassed the voices of all nine participants. It is significant also as the concept of resilience is widespread (but not without controversy) within the literature on mental health more generally, and specifically in regard to mental health and migration. An individual or community’s capacity for resilience must be considered within broader societal and ecological dimensions (Windle, 2011) and focusing intently on personal responsibility can “depoliticize” the factors and determinants that influence peoples’ capacities (Tanner et al., 2017, p. 1). Furthermore, the concept of resilience is valued, understood, and manifested differently in different cultures (Marttila et al., 2013; Raghavan et al., 2019). Cultivating resilience while allowing for the complexities of acculturation seems important, and community (rather than clinical) psychology may offer greater potential pathways for deconstructing and decolonising psychopathology through embracing cultural diversity (Rhodes & Langtiw, 2018).

Founding research related to the CHIME personal recovery framework in multi-ethnic Singapore (Lim et al., 2019) suggested hope to be the “cornerstone of personal recovery, fuelling one’s motivation to rebuild one’s life and self” (Discussion section, para. 6). This is likely related to regaining one’s internal locus of control, which has been found to be associated with higher levels of recovery (Morrison et al., 2013). Nonetheless, it is again crucial to underscore that agency can be confounded by a person’s wider social, economic, and political context (Marttila et al., 2013). This suggests that, like acculturation, resilience can be seen as a dynamic process of adaption in the midst of adversity (Luthar et al., 2000). Similarly, one can define recovery, as the process of resolving and overcoming difficulties and opportunities (van Weeghel et al., 2019). This was particularly pertinent for this study’s participants from CALD communities.

The affordances of the present study addressed how the power of narrative storytelling harnessed in the original participatory research enables direct engagement with people who are experts through experience. This helps to cultivate resilience, through enabling individuals who have been at the disempowered end of imbalanced power dynamics to regain their sense of control, and to empower them to tell and represent their stories in ways they want (Rhodes & De Jager, 2013). In this way, the participants’ narratives act as a root metaphor, that can allow one to enter the experience of another (Sarbin, 1986) adding value to one’s lived experience, and with the potential of generating greater empathy and compassion amongst the wider community. Ultimately, narrative storytelling offers opportunities for sense-making, reframing, connection, and recovery (Frank, 1995; Leamy et al., 2011; Robertson et al., 2020; Slade, 2009) in which culture plays an integral role (Kirmayer & Jarvis, 2019; Tse & Ng, 2014).

### Strengths and Limitations

The study’s strength is the deep exploration of nine individuals’ experiences of mental health recovery in the Australian context. The study extends and differentiates itself from the literature in two domains. Firstly, that of mental health recovery most often conducted in Anglophone countries with non CALD populations and frequently in accordance with the biomedical model. Secondly, it extends the literature exploring migrant and refugee mental health. In addition, the study makes use of data collected and produced during a digital storytelling (DST) participatory workshop, an inclusive method that offered individuals from CALD backgrounds the opportunity to share their lived experience with the public and wider research community (McDonough & Colucci, 2021).

This research sought to follow the aspirations set out by Lyons et al. (2013) based on four principles underlying feminist, multicultural, and social justice counselling: equity, access, participation, and harmony (Crethar et al., 2008). All participants in the initial study provided process consent (Smythe & Murray, 2000), meaning they can revoke their film at any point from the website where it is located online. All data have been transcribed verbatim from the films freely available online in the participants’ own words, with the intention that their stories are faithfully represented (Lyons et al., 2013). In keeping with secondary data analysis, the primary researcher of the current study did not have direct contact with any of the participants, and thus member checking did not occur (Iivari, 2018). However, she has attempted to develop her own cultural humility (Bogle et al., 2021) through consistent consideration of personal biases as they arose related to being an Australian citizen born in that country, with European roots.

As the study sample size was small, generalisability from the findings cannot be made. As purposive sampling (McDonough & Colucci, 2021) was utilised in the *Finding our way* project, it is possible participants felt strongly in some way about mental health recovery, Australia, and the benefits of narrative expression. As such, understandably, the narratives in this study do not represent the full spectrum of mental illness and recovery experiences. Inherent to the nature of a thematic analysis of transcriptions, valuable visual data, and auditory data other than direct narration do not form part of the analysis.

It is acknowledged that the participants/storytellers have lived and grown in the years since their films were created and thus, likely so too, have their relationships with recovery. As such, the current study is set in a particular time frame and reflects the storytellers and their narratives at the time of the workshop, rather than at the time of the present second layer of analysis of transcripts of the original data. The primary researcher understood that as the films were not an open dialogue or editable, there is a possible disconnection between the storytellers then, and their experiences and attitudes now (Gubrium et al., 2014; Woods et al., 2019). However, in 2017 the original authors conducted a follow up reflective study (McDonough & Colucci, 2021) involving more than half of the nine participants who expressed finding their participation “personally empowering and safe” (p. 18).

## Conclusion and Recommendations for Future Research

The findings of the current study contribute to the existing, albeit limited, body of research on how people from communities of difference including CALD, immigrant, refugee, or ethnic minority experience mental health recovery in diverse social, geographic, and cultural contexts. The themes arising from the self-authored narratives of the participants in this study shed light on the unique and yet not uncommon mental health recovery experiences of individuals of CALD backgrounds. The findings also offer support for utilising the extended CHIME framework with CALD communities and speaks to the diversity of mental health recovery experiences, and the varied ways in which people continue to shape and define their personhoods in relation to, or separate from, clinical definitions of recovery. Finally, it both evidences and furthers research on the intrinsic relationship between culture and mental health, from nine unique perspectives.

Given the increasing prevalence of migrant and refugee populations in many countries, and the concurrent impacts of trauma and mental ill-health compounded by experiences of marginalisation, studies of this kind further our capacity to understand the experiences of those affected. Mental health service provision can better target the needs of CALD communities through informed knowledge of what is dissonant as well as meaningful for people adjusting to living in complex and often overwhelming social contexts. This will include matters of mental health recovery during the COVID-19 pandemic, where migrant communities in Australia have experienced the harshest lockdowns, increased racism, and reduced opportunities for culturally safe healthcare (Couch, 2021; Plowman & Izzo, 2021). Future research could utilise similar participatory, visual methodologies to engage CALD communities and explore their experiences of the pandemic, with the knowledge that participatory arts activities have evidence for supporting recovery through social inclusion and promoting hope (Stickley et al., 2018). Furthermore, future research could also explore the particular recovery experiences and narratives of forced migrants as well as those expressed by people who identify with specific ethnocultural communities and/or other identity positions.

Research is developing on how to harness the potential of those with lived experience of mental health recovery in the context of the COVID-19 pandemic through questioning concepts of normality, stigma, social inequities, and broader determinants of health, as well as the role of the recovery framework in this context (Florence et al., 2021). In addition, experiences of recovery globally must continue to be explored through culturally appropriate avenues (Bayetti et al., 2016; Ricci et al., 2021) and in ways that actively seek to disrupt entrenched systems of privilege and power dynamics in research settings (Rose & Kalathil, 2019).

## Appendix 1: Participant transcripts

Participant one: It was horror. It was April 1992. War came to my home in Sarajevo. I had to leave with three sons; four, eight, 10. My husband stayed behind. I fled to Belgrade. Didn't know anyone there, but it was the only place to go. [music changes, slower] I asked help from Red Cross. The children enrolled in schools. Only a month later, my husband was killed by a neighbour. His childhood friend called me from Sarajevo to tell me. I was 38 years old. I was frightened, but I had to move forward for my children. Belgrade was hard so I made plans to take my children to Germany. It took three years to get there. I didn't know how to speak German but we made home there for three years. We left because the authorities wouldn't allow us to stay longer. We were refugees. We had to choose between going back to Yugoslavia, which was terror or America, Australia. It was cultural shock when I arrived in Australia. I had to retrain my thinking about everything. Everything my culture had expected of me was accomplished, was born, went through education, found a job, got married, have had children. But, that was the culture of the land that I came from. I needed to move in a science of mind. Learning how to live the life of my eventual new homeland. I remember I was walking the streets of Adelaide and constantly repeating the word “left” in my mind. Being on the left was a reminder that I was in Australia. When I was on right and bumping into someone, I knew I was on the wrong side of the street. I moved from survival mode into safe mode. When you are in survival mode the body is coping but once the threat stop, there is a calm environment, a still water. Oh, so much was suppressed. My body was in pain. Everything was out of balance. My body was telling me, sending a warning to me. I see this with other refugees stories. The pain of survival can transform into illness. And in 2001, I was diagnosed with breast cancer. My emotions were deeply embedded in both my body and subconscious. I was trapped in my emotions and terrified by my memories. Finally, everything popped, and I was able to understand, accept and respect. It helped me to connect and appreciate the good and bad in life. I still reflect and struggle with these feelings today. There are so many layers inside and you are never free. However, I'm a proud being and grateful of my transformation and the life I have.

Participant two: Dear little self, you were a blue child, always sick, always alone. In pain, fear, hunger. In perpetual darkness. Mum had to leave with your siblings. You must have felt unwanted, uncared for. I'm sorry. Please forgive Mum. She wouldn't have survived with five small children and you, still, a baby. [beeping – like a hospital monitor] Nannies took over. Fear and pain came back. [Twinkle twinkle choral in the background] You created dream people to hide behind. Someone to clothe and bathe you, feed you and rock you to sleep. Someone to chase away bad dreams. Someone to play with. Sometimes they felt more real than the real people. They kept you feeling safe and cared for. That was okay. [Choral music] As you grew older your needs changed, so you created different people to support you. You must have felt helpless when this cycle kept going on without any medical support or hope of rescue. You wanted to be heard, but were afraid to be seen, in case bad things happened again. In case you lost time, memories, and yourself. In case you found yourself in a bus, unable to breathe, frozen in fear. The journey to find that kind voice seemed daunting and you knew that scars fade but would always be there. Rejection, abandonment, pain and isolation. All the pressures in life, may tip your bucket and make you feel like hiding again, or crawling inside that bus again. You must have been lonely, being surrounded by more and more people, who did not know who you really were. I'm sorry. [Twinkle twinkle] But it's okay, we are finding real people to help us now. We will jump in a sailboat, with a camera, and capture our colours and our black. We will try to see the colours of life again today and/on the next day, and the next. [Choral music] It's okay little one. Life does not need to be perfect. Good enough is okay. Let the black mix with the colours and know that the picture looks even prettier that way.

Participant three: [aeroplane sound] [strange music] I grew up as a Karen boy in a refugee camp on the Thailand/Burma border with my family. As Karen refugees we had no rights, no freedom, no safety, no opportunities, no hope, or purpose for the future. There was poverty, danger and violence, discrimination, little education, nothing to do. Boredom and hopelessness led people to be involved with drugs, alcohol and fighting. Living in the camps was hard for families but also my Karen culture did not recognise the rights of children. Because of the war and poverty other reasons as a child you were not treated well. They did not teach us how to become someone. I grew up with ‘negatives’. My culture was restrictive and conservative. I could not express myself freely and there were strong expectations. UNHCR recognised me and my family as refugees and granted us humanitarian visas and arranged for us to be settled in Australia. Coming to Australia gave me my freedom, freedom to do what I want to do, and to be who I want to be. The opportunity to change my life. Having rights in Australia and opportunities showed me that I can make it here then I can make it anywhere. It helped me to build my confidence and to realise no one can stop me. [more positive music] I didn’t know anything about life before. My experiences here, as well as developing and maturing over the past five years has helped me change, learn, and grow stronger. Relationships with friends, other young people, teachers, workers, employers. Gaining knowledge here has helped me. Knowledge of life, of people, of myself. I have learnt many lessons in this new environment. I have opportunities to imagine and plan my future now. I just need to take my time and focus and keep the belief that I can be someone.

Participant four: [Easy, happy jazz music] I'm colours. I love my colours. I see life around me as colours and shapes. People don't make sense to me, no one makes sense to me. But I'm happy to see the colours. A small part of someone's dress or jewellery can connect me to them by the colour or the shape. Mostly I identify people by their clothing and the sound and colours that they indicate to me. If they take off that item of clothing it can be bit distracting. I'm happy that people remember me but I can't always return the favour. I like to put colours and shapes into a certain order which I see as a portal to my environment. It can be using bits and pieces I find in the street or trimmings passed down to me. Upscaling recycled material may not be the intention, but I am glad to be a part of it. I identify colours as a spectrum of life embellished with my understanding. When I put things together, I feel enlightened about the goodness around me. That joyful moment, I can't put into words. Codes, it's my own happiness. People might look at little curio [sic] embellishments that I make as a piece of art or something purpose. But to me it brings different colours shapes and sparkles together. That is my own little joy. I walk because I don't like public transport. Sometimes I find things I can use in my art, sometimes I get lost and I find difficult getting back to my house, so I use GPS or call someone familiar. If my phone is out of battery, I walk back along the tram tracks. I question myself. I ask am I different to other people? Many moons ago I would have been contributing to the community and living the concepts of average life. I swing between different planes, different dimensions. Colours are a pathway to reality for me. Counting buttons or beads help me to feel safe, so I don't feel like hiding under the doona or having so many showers in a day. A lot of professionals talk about healing and it's another confusing thing. I'm not keen about healing, it's more of a resolution. You have to make peace with the search for connectivity, a familiar voice that tells me I'm in a safe place and reassures me that I'm not flying away with the clouds.

Participant five: [calming, traditional Vietnamese music] I arrived in Australia in 1990 with my mum, my brother and sisters. We were sponsored by my father. I sometimes went to Vietnam to visit my grandma, because I love and miss her a lot. But now my grandma and my father passed away. After being in Australia for 4 years I started to be sick. For the first 10 years my sickness became severe. I was an inpatient in hospital for three years under the care of doctors, nurses, and medication. The government, through the Human Services, the carers, the social workers as well as the psychiatrist have helped me. Mostly the help of my family, particularly my mum’s love, have helped me to recover. Now, I can go to school twice a week to learn more of what I want to know. By joining the group with daily helpful activities, I can now cook, wash and clean up the home. I enjoy life with my hobbies such as singing (karaoke), I also am able to dress myself and put on makeup, to make myself feel satisfied. I’ve had excursions to other states… I went on a cruise recently to New Zealand with my mum and her friends. The trip was particularly enjoyable for me. I take this opportunity to express my appreciation to Australia and to my family for the help I received. The Australian people have been wonderful, some have been caring for us by giving us accommodation to get on with life. “I still call Australia home” just like Peter Allan in his song.

Participant six: [Calm, piano music] Faith is the reason why even in pain, I smile. In confusion, I understand. In betrayal, I trust. In fear, I continue to fight. I never forget the day I left my family in a civil war country, Sri Lanka. The fear of never seeing them again, every day and night I thought about them. So many tears. I love my family. Arrived in Australia, I was trying to survive. It was a cultural shock. Years passed by. I finally found my lifetime partner. I got married and had four beautiful children, three boys and a one girl. I adore[d?] them dearly. We move[d?] from one city to another. Our children were very young. Our daughter only a couple of month old. I start a job and full-time study. It didn't go well for me. The darkness start creeping into my life. As a mother and a wife, I have responsibilities. I tried my best to fulfill those responsibilities in a foreign country with no family, no help, just my husband and I, our children and faith in God. My husband saw the warning signs, the dark clouds appearing in my life. He told me to slow down. Unfortunately, darkness already got hold of me. No help?. I drift away from God, from my faith, even though I drift away from my god, he never let go. He reached down from on high and took hold of me. He dreamed? me out of deep water. True peace does not come as a result of eliminating sorrows and disappointments, it comes as a result of one thing, and that is an intimate relationship with the Lord Jesus Christ. To me, he is where anxiety ends, and peace begins. Faith is the significant element in my recovery journey. I'm thanking God for my family and friends who prayed for my recovery. My anchor was my husband and my children and my family overseas. Challenges are what make life interesting and overcoming them is what make life meaningful. I have had many disappointments but I learned from all of my experiences. Now I love helping others through their own recovery journeys. My Heavenly Father is protecting me. I have joy inside me. One thing, nobody cannot take that away from me.

Participant seven: [earthy, psychedelic music] It all began in the early 90 s with the legend of Homopia, a healing story of how a planet parallel to the earth begins to move down a path of insanity. How the spirits of nature work closely with humans to heal the brokenness of the ancient archetype, the madman. Then came the journey of the madman where the madman becomes the centre of a vortex of insanity, on a planetary scale. The beings of the planet go in search of their Holy Grail, the ultimate medicine, Artraham. If they can find the ingredients and make the most powerful healing medicine ever created, yet Atraham still remains undiscovered. [Psychedelic Superhero] My graphic novel brings the Light Warrior Psychiatric Superhero’s deep inner struggles out into the open, in a highly imaginative and haunting saga. I'm endeavouring to visually display this archetypal journey from the state of being cursed to one of liberation, and through the madman's trials and tribulations, illustrate how to become his own healer and healer redeemer. The graphic novel is a story but the experiences of the Psychiatric Superhero and the strategies he employs to triumph over evil are drawn from the knowledge and practices I have developed in my daily practice of managing schizophrenia over many years. Mantras are a colourful, powerful device in my graphic novels. I use them to take back my power from the negative. For two hours every day I go into the workshop of the mind and practice. Like playing the scales of the piano. Whatever the negative behind the voices is, it is a bully and it responds to you telling it what for. It requires persistence but with practice you'll succeed. For mentally ill people that's what it is. I decided a long time ago that I was going to outrun the voices. I would drown them out with my own inner voice. My core mantra, “I take back my infinite power from the negative, even here even now”, will be woven into the fabric of the tale. I have a dream that Psychiatric Superhero will one day become a powerful computer game that will give all who play it valuable insight into the human psyche and how to live free of the modern scourge of recreational drugs and mental illness.

Participant eight: [Chinese? Music] After my mental breakdown, I found I was lost and couldn’t concentrate on anything. I started to make origami fish. I used red pockets to make fish to calm myself down. I started making fish whenever I could. In the house there were fish everywhere, all over my table, chairs and even the floor. Then I thought, where should they go? It was Christmas time, I took my fish and jumped on the city trams. I gave my fish to the tram drivers and passengers and said “Merry Christmas”. They were all surprised. Then I also began teaching my neighbours to make fish. In 2003, I learned to be a laughter leader. At that time I found out this is the only exercise I can do at anytime, anywhere. Even in my situation, I could still laugh. Laughing makes me happy and it also brings happiness to others. Now I still go to different communities to bring my laugh to both old and young. Once a month I cook a welcome lunch the new residents to move into the housing estate [sic]. They are from different backgrounds Chinese, African, Aussie. I also cook for different community events and at the Chinese Buddhist temple. People say my K.L.C. (Kim Ling Chicken) is better than K.F.C. I love to cook. Every time I see people leaving an empty plate, it gives me great pleasure and a big reward. Recently I started learning to grow seasonable vegetables. It makes me very happy to share food that I have grown and cooked myself. HO! HO! HA! HA! HA! HO! HO! HA! HA! HA! HAHAHAHAHA….!! HAHAHAHAHA….!!

Participant nine: [orchestral music] The purpose of my life is to experience life, to do what I love, and to have a balance. My cultural background is of Greek heritage. Born in Melbourne, Australia. My upbringing has conditioned me as a female to care for my parents. This role over the years has inhibited my growth. However, through its challenges, I have learned to put myself first, and am now a more empowered and resilient person. In year 12, I chose the medium of ceramics. I was interested in working with the three-dimensional form. It was my art teacher who persuaded me to apply for a degree in Fine Arts, majoring in ceramics. I studied and graduated as a formal visual artist from RMIT Melbourne. I exhibited and curated my work around Melbourne galleries. I also completed a postgraduate diploma in Arts Curatorship and Museum Management. And I developed my own decoupage technique. Then in 2011, I became represented by *archsign* style gallery in Victoria. I have a passion for collecting. I collect books. Inspired by my interest in classical literature such as Lewis Carroll’s Alice in Wonderland. Fairy tales. Film. Art. And self-help. I also collect films and television series’ and have over 300 DVDs to my collection. I collect art from other artists, some of which are friends, but my prized possession is an Arthur Boyd etching. Children are important to me and I believe they keep me young. Paul is 14. He loves Hawthorn and gadgets. Gina is 10. She loves guineapigs and making music videos. Stephan is eight. He likes the Hawks and reading. Christina is six. She loves swimming and drama. Katherine is eight. She loves to work on projects and being artistic. Helena is six. She is very mischievous and inquisitive. James and Andrea are twins. They are almost two. He loves the outdoors and activities, and she loves dancing and cuddles. For me, family is key to wellness. My family and extended family are a very important part of my life.

## References

[CR1] Abdi SM, Miller AB, Agalab NY, Ellis BH (2021). Partnering with refugee communities to improve mental health access: Going from “why are they not coming” to “what can I (we) do differently?”. Cultural Diversity and Ethnic Minority Psychology..

[CR2] Adame AL, Hornstein GA (2006). Representing madness: How are subjective experiences of emotional distress presented in first-person accounts?. The Humanistic Psychologist.

[CR3] Alim M, Due C, Strelan P (2021). Relationship between experiences of systemic injustice and wellbeing among refugees and asylum seekers: A systematic review. Australian Psychologist.

[CR4] Anderson Clarke LM, Warner B (2016). Exploring recovery perspectives in individuals diagnosed with mental illness. Occupational Therapy in Mental Health.

[CR5] Antonovsky A (1967). Aspirations, class and racial-ethnic membership. The Journal of Negro Education.

[CR6] Antonovsky A (1979). Health, stress, and coping.

[CR7] Attride-Stirling J (2001). Thematic networks: An analytic tool for qualitative research. Qualitative Research.

[CR8] Bayetti, C., Jadhav, S., & Jain, S. (2016). The re-covering self: A critique of the recovery-based approach in India’s mental health care. Disability and the Global South, 3(1), 889–909. https://disabilityglobalsouth.files.wordpress.com/2012/06/dgs-03-01-04.pdf

[CR9] Berger, P. L., & Luckmann, T. (1966). The social construction of reality: A treatise in the sociology of knowledge. Anchor.

[CR10] Berry JW, Bhugra D, Bhui K (2018). Acculturation and identity. Textbook of Cultural Psychiatry.

[CR11] Berry JW, Hou F (2019). Multiple belongings and psychological well-being among immigrants and the second generation in Canada. Canadian Journal of Behavioural Science/revue Canadienne Des Sciences Du Comportement.

[CR12] Bhavsar V, Zhang S, Bhugra D (2019). Conceptualizing globalization for mental health research. International Journal of Social Psychiatry.

[CR13] Bhugra D, Becker MA (2005). Migration, cultural bereavement and cultural identity. World Psychiatry: Official Journal of the World Psychiatric Association (WPA).

[CR14] Bhugra D, Wojcik W, Gupta S, Bhugra D, Gupta S (2010). Cultural bereavement, culture shock and culture conflict: Adjustments and reactions. Migration and mental health.

[CR15] Bhugra D, Watson C, Ventriglio A (2021). Migration, cultural capital and acculturation. International Review of Psychiatry.

[CR16] Bhui K, Black T, Bhugra D, Gupta S (2010). Identity, idioms and inequalities: Providing psychotherapies for South Asian women. Migration and mental health.

[CR17] Blackwell D (2005). Counselling and Psychotherapy with Refugees.

[CR18] Bogic, M., Njoku, A., & Priebe, S. (2015). Long-term mental health of war-refugees: A systematic literature review. BMC International Health and Human Rights, 15, Article 29. 10.1186/s12914-015-0064-9 10.1186/s12914-015-0064-9 PMC4624599 26510473

[CR19] Bogle A, Rhodes P, Hunt C (2021). Cultural humility and decolonial practice: Narratives of therapists’ lives. Clinical Psychologist.

[CR20] Bolton D, Gillet G (2019). The Biopsychosocial model of health and disease. Amsterdam University Press.

[CR21] Bonnington O, Rose D (2014). Exploring stigmatisation among people diagnosed with either bipolar disorder or borderline personality disorder: A critical realist analysis. Social Science & Medicine.

[CR22] Bracken, P., Fernando, S., Alsaraf, S., Creed, M., Double, D., Gilberthorpe, T., Hassan, R., Jadhav, S., Jeyapaul, P., Kopua, D., Parsons, M., Rodger, J., Summerfield, D., Thomas, P., & Timimi, S. (2021). Decolonising the medical curriculum: Psychiatry faces particular challenges*. *Anthropology & Medicine, 1–9. Advance online publication. 10.1080/13648470.2021.1949892 10.1080/13648470.2021.1949892 34282672

[CR23] Braun V, Clarke V (2006). Using thematic analysis in psychology. Qualitative Research in Psychology.

[CR24] Braun V, Clarke V, Terry G, Rohleder P, Lyons AC (2014). Thematic analysis. Qualitative research in clinical and health psychology.

[CR25] Braun V, Clarke V, Hayfield N (2019). ‘A starting point for your journey, not a map’: Nikki Hayfield in conversation with Virginia Braun and Victoria Clarke about thematic analysis. Qualitative Research in Psychology.

[CR26] Braun, V., & Clarke, V. (2020). One size fits all? What counts as quality practice in (reflexive) thematic analysis? Qualitative Research in Psychology, Advance online publication10.1080/14780887.2020.1769238

[CR27] Brijnath B (2015). Applying the CHIME recovery framework in two culturally diverse Australian communities: Qualitative results. International Journal of Social Psychiatry.

[CR28] Bulik KJD, Colucci E (2019). Refugees, resettlement experiences and mental health: A systematic review of case studies. Jornal Brasileiro De Psiquiatria.

[CR29] Casey B, Webb M (2019). Imaging journeys of recovery and learning: A participatory arts-based inquiry. Qualitative Health Research.

[CR30] Colucci E, Bhui K (2015). Arts, media and mental health. World Cultural Psychiatry Research Review.

[CR31] Colucci E, McDonough S, Lo V, Berry C, Liping G (2020). Recovering from mental illness and suicidal behaviour in a culturally diverse context: The use of digital storytelling. Film and the Chinese medical humanities.

[CR32] Couch J (2021). ‘We were already strong’: Young refugees, challenges and resilience during COVID-19. Journal of Social Inclusion.

[CR33] Crethar HC, Rivera ET, Nash S (2008). In search of common threads: Linking multicultural, feminist, and social justice counseling paradigms. Journal of Counseling & Development.

[CR34] Crowe M (2006). Psychiatric diagnosis: Some implications for mental health nursing care. Journal of Advanced Nursing.

[CR35] Crowe M, Inder M, Porter R (2015). Conducting qualitative research in mental health: Thematic and content analyses. Australian & New Zealand Journal of Psychiatry.

[CR36] Davidson L (2019). Is “Personal recovery” a useful measure of clinical outcome?. Psychiatric Services.

[CR37] Davidson, L., Rowe, M., DiLeo, P., Bellamy, C., & Delphin-Rittmon, M. (2021). Recovery-oriented systems of care: A Perspective on the past, present, and future. Alcohol Research: Current Reviews, 41(1), Article 9. 10.35946/arcr.v41.1.09 10.35946/arcr.v41.1.09 PMC8336784 34377618

[CR38] De Montfort University. (2021, January 4). *A photographic exploration of Romanian mental health service users’ experience of recovery* [Video]. YouTube. https://www.youtube.com/watch?v=Rixm9t3pq-o&feature=youtu.be

[CR39] De Vecchi N, Kenny A, Dickson-Swift V, Kidd S (2016). How digital storytelling is used in mental health: A scoping review. International Journal of Mental Health Nursing.

[CR40] Deegan P (1996). Recovery as a journey of the heart. Psychiatric Rehabilitation Journal.

[CR41] Deegan P (2007). The lived experience of using psychiatric medication in the recovery process and a shared decision-making program to support it. Psychiatric Rehabilitation Journal.

[CR42] DeSantis L, Ugarriza DN (2000). The concept of theme as used in qualitative nursing research. Western Journal of Nursing Research.

[CR301] Diocera, D., & Colucci, E. (in press). Undertaking mental health and suicide prevention research with people from immigrant and refugee backgrounds

[CR43] Doroud N, Fossey E, Fortune T, Brophy L, Mountford L (2022). A journey of living well: A participatory photovoice study exploring recovery and everyday activities with people experiencing mental illness. Journal of Mental Health.

[CR44] Drake RE, Whitley R (2014). Recovery and severe mental illness: Description and analysis. Canadian Journal of Psychiatry..

[CR45] Duff C (2016). Atmospheres of recovery: Assemblages of health. Environment and Planning A.

[CR46] Eaton J (2019). Rebalancing power in global mental health. International Journal of Mental Health.

[CR47] Edge D, Bhugra D, Castle D, Abel K (2016). Ethnic and cultural effects on mental healthcare for women. Comprehensive women's mental health.

[CR48] Enticott, J. C., Shawyer, F., Vasi, S., Buck, K., Cheng, I. H., Russell, G., Kakuma, R., Minas, H., & Meadows, G. (2017). A systematic review of studies with a representative sample of refugees and asylum seekers living in the community for participation in mental health research. BMC Medical Research Methodology, 17, Article 37. 10.1186/s12874-017-0312-x 10.1186/s12874-017-0312-x PMC5335792 28253851

[CR49] Fernando, S. (2017). Institutional racism in psychiatry and clinical psychology: Race matters in mental health*.* Palgrave Macmillan

[CR50] Florence AC, Miller R, Bellamy C, Bernard P, Bien C, Atterbury K, Bragg C, Diaz A, Gardien E, Guy K, Hansen C, Maclean K, Milton B, Nelson L, Samoskevich JJ, Smith S, Stanojlovic M, Wexler T, Zorzanelli R, Davidson L (2021). When reality breaks from us: Lived experience wisdom in the Covid-19 era. Psychosis.

[CR51] Fozdar F, Hartley L (2013). Civic and ethno belonging among recent refugees to Australia. Journal of Refugee Studies.

[CR52] Frank, A. W. (1995). *The wounder storyteller: Body, illness, and ethics.* The University of Chicago Press

[CR53] Glynn SM, Cohen AN, Dixon LB, Niv N (2006). The potential impact of the recovery movement on family interventions for schizophrenia: Opportunities and obstacles. Schizophrenia Bulletin.

[CR54] Gómez-Carrillo A, Lencucha R, Faregh N, Veissière S, Kirmayer LJ (2020). Engaging culture and context in mhGAP implementation: Fostering reflexive deliberation in practice. BMJ Global Health.

[CR55] Graham R, Clarke V (2021). Staying strong: Exploring experiences of managing emotional distress for African Caribbean women living in the UK. Feminism & Psychology.

[CR56] Gubrium AC, Hill AL, Flicker S (2014). A situated practice of ethics for participatory visual and digital methods in public health research and practice: A focus on digital storytelling. American Journal of Public Health.

[CR57] Harper D, Speed E (2012). Uncovering recovery: The resistible rise of recovery and resilience. Studies in Social Justice.

[CR58] Herman, J. (1992). *Trauma and recovery: The Aftermath of violence*. Basic Books.

[CR59] Hopper K (2007). Rethinking social recovery in schizophrenia: What a capabilities approach might offer. Social Science & Medicine.

[CR60] Hwang W, Myers H, Abekim J, Ting J (2008). A conceptual paradigm for understanding culture’s impact on mental health: The cultural influences on mental health (CIMH) model. Clinical Psychology Review.

[CR61] Ida DJ (2007). Cultural competency and recovery within diverse populations. Psychiatric Rehabilitation Journal.

[CR62] Iivari N (2018). Using member checking in interpretive research practice. Information Technology & People.

[CR63] Inman AG, Ladany N, Constantine MG, Morano CK (2001). Development and preliminary validation of the Cultural Values Conflict Scale for South Asian women. Journal of Counseling Psychology.

[CR64] Jacobson N, Farah D (2012). Recovery through the lens of cultural diversity. Psychiatric Rehabilitation Journal.

[CR65] Jadhav, S. (2009). What is cultural validity and why is it ignored? In S. V. D. Geest & M. Tankink (Eds.), *Theory and action: Essays for an anthropologist* (pp. 92–96). AMB Diemen 2009

[CR66] Jannesari S, Molyneaux E, Lawrence V (2019). What affects the mental health of people seeking asylum in the UK? A narrative analysis of migration stories. Qualitative Research in Psychology.

[CR67] Kaiser BN, Kohrt BA, Keys HM, Khoury NM, Brewster ART (2013). Strategies for assessing mental health in Haiti: Local instrument development and transcultural translation. Transcultural Psychiatry.

[CR68] Kaiser BN, Varma S, Carpenter-Song E, Sareff R, Rai S, Kohrt BA (2020). Eliciting recovery narratives in global mental health: Benefits and potential harms in service user participation. Psychiatric Rehabilitation Journal.

[CR69] Kalathil, J., Bhakta, R., Daniel, O., Joseph, D., & Trivedi, P. (2011). *Recovery and resilience: African, African-Caribbean and South Asian women’s narratives of recovering from mental distress*. Mental Health Foundation.

[CR70] Kale A, Kindon S, Stupples P (2018). ‘I am a New Zealand citizen now—This is my home’: Refugee citizenship and belonging in a post-colonizing Country. Journal of Refugee Studies.

[CR71] Katsifis, V., Oehm, D., Di Mezza, S. & Cranfield, D. (2017). Vox pop: Cultural diversity and mental health. *New Paradigm.* Winter, 27–29.

[CR72] Kirkbride J, Jones P, Bhugra D, Gupta S (2010). Epidemiological aspects of migration and mental illness. Migration and mental health.

[CR73] Kirmayer LJ, Jarvis GE (2019). Culturally responsive services as a path to equity in mental healthcare. Healthcare Papers.

[CR74] Kleinman A (1978). Concepts and a model for the comparison of medical systems as cultural systems. Social Science & Medicine Part b: Medical Anthropology.

[CR75] Knifton L (2012). Understanding and addressing the stigma of mental illness with ethnic minority communities. Health Sociology Review.

[CR76] LaMarre, A., & Rice, C. (2016). Embodying critical and corporeal methodology: Digital storytelling with young women in eating disorder recovery. Forum Qualitative Sozialforschung, 17(2). 10.17169/fqs-17.2.2474

[CR77] Lamb K, Boyles J (2017). Recovery and reconnection. Psychological therapies for survivors of torture: A human-rights approach with people seeking asylum.

[CR78] Lawrence V, McCombie C, Nikolakopoulos G, Morgan C (2021). Navigating the mental health system: Narratives of identity and recovery among people with psychosis across ethnic groups. Social Science & Medicine.

[CR79] Leamy M, Bird V, Boutillier CL, Williams J, Slade M (2011). Conceptual framework for personal recovery in mental health: Systematic review and narrative synthesis. British Journal of Psychiatry.

[CR154] Lemke T (2001). The birth of bio-politics: Michel Foucault's lecture at the Collège de France on neo-liberal Governmentality. Econ Soc.

[CR80] Lim M, Li Z, Xie H, Tan BL, Lee J (2019). An Asian study on clinical and psychological factors associated with personal recovery in people with psychosis. BMC Psychiatry.

[CR81] Lincoln YS (1995). Emerging criteria for quality in qualitative and interpretive research. Qualitative Inquiry.

[CR82] Llewellyn-Beardsley J, Rennick-Egglestone S, Callard F, Crawford P, Farkas M, Hui A, Manley D, McGranahan R, Pollock K, Ramsay A, Sælør KT, Wright N, Slade M (2020). Characteristics of mental health recovery narratives: Systematic review and narrative synthesis. PLoS ONE.

[CR83] Lo MCM (2010). Cultural brokerage: Creating linkages between voices of lifeworld and medicine in cross-cultural clinical settings. Health: an Interdisciplinary Journal for the Social Study of Health Illness and Medicine.

[CR84] Luthar SS, Cicchetti D, Becker B (2000). The construct of resilience: A critical evaluation and guidelines for future work. Child Development.

[CR85] Lyons HZ, Bike DH, Ojeda L, Johnson A, Rosales R, Flores LY (2013). Qualitative research as social justice practice with culturally diverse populations. Journal for Social Action in Counseling & Psychology.

[CR86] Marsella AJ (2010). Ethnocultural aspects of PTSD: An overview of concepts, issues, and treatments. Traumatology.

[CR87] Marsella, A. J. (1980). Depressive experience and disorder across culture. In H. C. Triandis & J. G. Fraguns (Eds.), *Handbook of cross-cultural psychology* (Vol. 6, pp. 237–289). Allyn and Bacon.

[CR303] Marttila A, Johansson E, Whitehead M, Burström B (2015). Keep going in adversity – using a resilience perspective to understand the narratives of long-term social assistance recipients in Sweden. International Journal for Equity in Health.

[CR88] Matsuoka AK (2015). Ethnic/Racial minority older adults and recovery: Integrating stories of resilience and hope in social work. The British Journal of Social Work.

[CR89] McDonough S, Colucci E (2021). People of immigrant and refugee background sharing experiences of mental health recovery: Reflections and recommendations on using digital storytelling. Visual Communication.

[CR90] Meadows, E. (2017). Navigating the language maze: Mental health in the context of migration. *New Paradigm*. Winter, 27–29

[CR91] Metzl, J. M. (2009). *The protest psychosis: How schizophrenia became a black disease*. Beacon Press

[CR92] Milasan, L. H., Bingley, A. F., & Fisher, N. R. (2020). The big picture of recovery: A systematic review on the evidence of photography-based methods in researching recovery from mental distress. Arts & Health, Advance online publication10.1080/17533015.2020.1855453 10.1080/17533015.2020.1855453 33252304

[CR93] Minas H, Bhugra D, Bhui K (2018). Developing effective mental health services for multicultural societies. Textbook of cultural psychiatry.

[CR94] Minas H, Kakuma R, Too L, Vayani H, Orapeleng S, Prasad-Ildes R, Turner G, Procter N, Oehm D (2013). Mental health research and evaluation in multicultural Australia: Developing a culture of inclusion. International Journal of Mental Health Systems.

[CR95] Mishler EG (1984). The discourse of medicine: Dialectics of medical interviews.

[CR96] Morgan, A., Felton, A., Fulford, B., Kalathil, J., & Stacey, G. (2016). Values and ethics in mental health: An exploration for practice. Macmillan International Higher Education.

[CR97] Morrison AP, Shryane N, Beck R, Heffernan S, Law H, McCusker M, Bentall RP (2013). Psychosocial and neuropsychiatric predictors of subjective recovery from psychosis. Psychiatry Research.

[CR98] Morrow, V., Boddy, J., & Lamb, R. (2014, April). *The ethics of secondary data analysis: Learning from the experience of sharing qualitative data from young people and their families in an international study of childhood poverty*. Institute of Education, University of London. https://assets.publishing.service.gov.uk/media/57a089f6ed915d3cfd0004f4/novella_morrow-et-al_wp.pdf

[CR99] Morrow M, Weisser J (2012). Towards a social justice framework of mental health recovery. Studies in Social Justice.

[CR100] Mousaferiadis, P. (n.d.). *Why ‘Culturally and Linguistically Diverse’ Has Had Its Day*. Diversity Atlas. https://www.diversityatlas.com.au/heres-why-cald-has-had-its-day/

[CR101] Movie-ment. (2014). *Participatory digital stories on journeys of recovery, Finding our way*. https://movie-ment.org/findingourway/

[CR102] Nazroo JY, Bhui KS, Rhodes J (2020). Where next for understanding race/ethnic inequalities in severe mental illness? Structural, interpersonal and institutional racism. Sociology of Health & Illness.

[CR103] Nurser KP, Rushworth I, Shakespeare T, Williams D (2018). Personal storytelling in mental health recovery. Mental Health Review Journal.

[CR104] O’Hagan M, Reynolds P, Smith C (2012). Recovery in New Zealand: an evolving concept?. International Review of Psychiatry.

[CR105] Pelletier JF, Davidson L, Giguère CÉ, Franck N, Bordet J, Rowe M (2020). Convergent and concurrent validity between clinical recovery and personal-civic recovery in mental health. Journal of Personalized Medicine.

[CR106] Persaud A, Tribe R, Bhugra D, Bhui K, Rathod S, Willis J (2019). Careif position statement on migration and mental health. World Cultural Psychiatry Research Review.

[CR107] Petros R, Solomon P, Linz S, DeCesaris M, Hanrahan NP (2016). Autovideography: The lived experience of recovery for adults with serious mental illness. Psychiatric Quarterly.

[CR108] Piat M, Seida K, Sabetti J (2017). Understanding everyday life and mental health recovery through CHIME. Mental Health and Social Inclusion.

[CR109] Plowman, M., & Izzo, S. (2021, June). *Recommendations for a culturally responsive mental health system*. Ethnic Communities’ Council of Victoria and Victorian Transcultural Mental Health. https://vtmh.org.au/wp-content/uploads/2021/06/Recommendations-for-a-Culturally-Responsive-Mental-Health-System-Report_ECCV_VTMH_June-2021.pdf

[CR110] Price-Robertson R, Obradovic A, Morgan B (2016). Relational recovery: Beyond individualism in the recovery approach. Advances in Mental Health.

[CR111] Procter N, Babakarkhil A, Baker A, Ferguson M, Procter N, Hamer HP, McGarry D, Wilson RL, Froggatt T (2014). Mental health of people of immigrant and refugee backgrounds. Mental Health: A person-centred approach.

[CR112] Provencher HL, Keyes CLM, Keyes CLM (2013). Recovery: A complete mental health perspective. Mental Well-Being.

[CR113] Pūras, D. (2017). Report of the Special Rapporteur on the right of everyone to the enjoyment of the highest attainable standard of physical and mental health. United Nations Human Rights Council. http://undocs.org/A/HRC/35/21

[CR114] Pūras, D. (2020). Right of everyone to the enjoyment of the highest attainable standard of physical and mental health: Report of the Special Rapporteur on the right of everyone to the enjoyment of the highest attainable standard of physical and mental health. United Nations Human Rights Council. http://undocs.org/A/HRC/44/48

[CR115] Puvimanasinghe T, Denson LA, Augoustinos M, Somasundaram D (2014). “Giving back to society what society gave us”: Altruism, coping, and meaning making by two refugee communities in South Australia. Australian Psychologist.

[CR116] Raghavan R, Coope J, Jamwal S, Pendse T (2019). Reflections on the use of mental health resilience concepts in migration and global mental health. International Journal of Mental Health.

[CR117] Rhodes P, de Jager A (2013). Narrative studies of recovery: A critical resource for clinicians. Clinical Psychologist.

[CR118] Rhodes P, Langtiw C (2018). Why clinical psychology needs to engage in community-based approaches to mental health. Australian Psychologist.

[CR119] Ricci ÉC, Leal EM, Davidson L, Costa M (2021). Narratives about the experience of mental illness: The recovery process in Brazil. Psychiatric Quarterly.

[CR120] Robertson S, Carpenter D, Donovan-Hall M, Bartlett R (2020). Using lived experience to develop a personal narrative workshop programme in order to aid mental health recovery. Journal of Mental Health.

[CR121] Rose D (2014). The mainstreaming of recovery. Journal of Mental Health.

[CR122] Rose D, Kalathil J (2019). Power, privilege and knowledge: The untenable promise of co-production in mental “Health”. Frontiers in Sociology.

[CR123] Rose E, Bingley A, Rioseco M, Lamb K (2018). Art of recovery: Displacement, mental health, and wellbeing. Arts.

[CR124] Ruggiano N, Perry TE (2019). Conducting secondary analysis of qualitative data: Should we, can we, and how?. Qualitative Social Work.

[CR125] Sarbin, T. R. (1986). Narrative psychology: The storied nature of human conduct. Praeger

[CR126] Sawrikar, P., & Katz, I. (2009). How useful is the term ‘culturally and linguistically diverse’ (CALD) in Australian research, practice and policy discourse? Social Policy Research Centre

[CR127] Slade M (2009). Personal recovery and mental illness: A guide for mental health professionals.

[CR128] Slade M, Leamy M, Bacon F, Janosik M, Le Boutillier C, Williams J, Bird V (2012). International differences in understanding recovery: Systematic review. Epidemiology and Psychiatric Sciences.

[CR129] Slade M, Amering M, Farkas M, Hamilton B, O'Hagan M, Panther G, Perkins R, Shepherd G, Tse S, Whitley R (2014). Uses and abuses of recovery: Implementing recovery-oriented practices in mental health systems. World Psychiatry.

[CR130] Slade, M., Bird, V., Chandler, R., Clarke, E., Craig, T., Larsen, J., Lawrence, V., Le Boutillier, C., Macpherson, R., McCrone, P., Pesola, F., Riley, G., Shepherd, G., Tew, J., Thornicroft, G., Wallace, G., Williams, J., & Leamy, M. (2017) *REFOCUS: developing a recovery focus in mental health services in England.* Institute of Mental Health. http://eprints.nottingham.ac.uk/id/eprint/47617

[CR131] Slattery M, Attard H, Stewart V, Roennfeldt H, Wheeler AJ (2020). Participation in creative workshops supports mental health consumers to share their stories of recovery: A one-year qualitative follow-up study. PLoS ONE.

[CR132] Smythe WE, Murray MJ (2000). Owning the story: Ethical considerations in narrative research. Ethics & Behavior.

[CR133] Stickley T, Wright N, Slade M (2018). The art of recovery: Outcomes from participatory arts activities for people using mental health services. Journal of Mental Health.

[CR134] Stuart SR, Tansey L, Quayle E (2016). What we talk about when we talk about recovery: A systematic review and best-fit framework synthesis of qualitative literature. Journal of Mental Health.

[CR135] Summerfield D, Rosen GM (2004). Cross-cultural perspectives on the medicalization of human suffering. Posttraumatic stress disorder: Issues and controversies.

[CR300] Summerfield D (2012). Afterword: Against “global mental health”. Transcultural Psychiatry.

[CR136] Synergi Collaborative Centre. (2019, February). The importance of participatory methods to research and system change. https://synergicollaborativecentre.co.uk/wp-content/uploads/2017/11/Importance-of-participatory-methods-briefing-paper.pdf

[CR137] Talwar S (2010). An intersectional framework for race, class, gender, and sexuality in art therapy. Art Therapy.

[CR138] Tang L (2019). The double hazard in recovery journey: The experiences of UK Chinese users of mental health services. The International Journal of Social Psychiatry.

[CR139] Tanner, T., Bahadur, A., & Moench, M. (2017, September). Challenges for resilience policy and practice. Overseas Development Institute. https://cdn.odi.org/media/documents/11733.pdf

[CR140] Thorne, S. (1994). Secondary analysis in qualitative research: Issues and implications. In J. M. More (Ed.), *Critical issues in qualitative research methods* (1st ed., pp. 263–279). Sage.

[CR141] Tracy SJ (2010). Qualitative Quality: Eight “Big-Tent” criteria for excellent qualitative research. Qualitative Inquiry.

[CR142] Tse S, Ng RMK (2014). Applying a mental health recovery approach for people from diverse backgrounds: The case of collectivism and individualism paradigms. Journal of Psychosocial Rehabilitation and Mental Health.

[CR143] van Weeghel J, van Zelst C, Boertien D, Hasson-Ohayon I (2019). Conceptualizations, assessments, and implications of personal recovery in mental illness: A scoping review of systematic reviews and meta-analyses. Psychiatric Rehabilitation Journal.

[CR144] Vansteenkiste T, Morrens M, Westerhof GJ (2021). Images of Recovery: A PhotoVoice study on visual narratives of personal recovery in persons with serious mental illness. Community Mental Health Journal.

[CR145] Virdee G, Frederick T, Tarasoff LA, McKenzie K, Davidson L, Kidd SA (2017). Community participation within the context of recovery: Multiple perspectives on South Asians with schizophrenia. International Journal of Culture and Mental Health.

[CR146] Watson C, Bhugra D (2020). Transcultural Psychiatric Assessment. Medicine.

[CR147] Wei K, Chopra P, Strehlow S, Stow M, Kaplan I, Szwarc J, Minas H (2021). The capacity-building role of community liaison workers with refugee communities in Victoria, Australia. International Journal of Mental Health Systems.

[CR148] White RG, Imperiale MG, Perera E (2016). The Capabilities Approach: Fostering contexts for enhancing mental health and wellbeing across the globe. Globalization and Health.

[CR149] Whitley R, Sitter KC, Adamson G, Carmichael V (2021). A meaningful focus: Investigating the impact of involvement in a participatory video program on the recovery of participants with severe mental illness. Psychiatric Rehabilitation Journal.

[CR150] Windle G (2011). What is resilience? A review and concept analysis. Reviews in Clinical Gerontology.

[CR151] Woods, A., Hart, A., & Spandler, H. (2019). The recovery narrative: Politics and possibilities of a genre. *Culture, Medicine and Psychiatry*, Advance online publication10.1007/s11013-019-09623-y 10.1007/s11013-019-09623-y PMC9034998 30895516

[CR155] Wong SHM, Gishen F, Lokugamage AU (2021). Decolonising the medical curriculum: Humanising Medicine through epistemic pluralism, cultural safety and critical consciousness. London Rev Edu.

[CR152] Ysseldyk R, Haslam SA, Haslam C (2013). Abide with me: Religious group identification among older adults promotes health and well-being by maintaining multiple group memberships. Aging & Mental Health.

[CR153] Ziaian T, Puvimanasinghe T, Miller E, de Anstiss H, Esterman A, Dollard M (2021). Identity and belonging: Refugee youth and their parents’ perception of being Australian. Australian Psychologist.

